# Nonalcoholic Fatty Liver Disease: Study of Demographic and Predictive Factors

**DOI:** 10.5005/jp-journals-10018-1119

**Published:** 2015-01-06

**Authors:** Bimal Chandra Shil, Madhusudan Saha, Faruque Ahmed, Swapan Chandra Dhar

**Affiliations:** 1 Department of Gastroenterology, Sir Salimullah Medical College, Dhaka, Bangladesh; 2 Department of Gastroenterology, North East Medical College, Sylhet, Bangladesh; 3 Department of Gastroenterology, Dhaka Medical College, Dhaka, Bangladesh

**Keywords:** NAFLD, Prevalence, Risk factors.

## Abstract

**Background:**

Nonalcoholic fatty liver disease (NAFLD) represents a spectrum of liver disease characterized by excess of fat in liver which ranges from simple steatosis to nonalcoholic steato-hepatitis (NASH), cirrhosis and hepatocellular carcinoma (HCC) in the absence of excessive alcohol consumption.

**Materials and methods:**

The study was carried out in 216 with serologically defined fatty liver. They underwent detailed history evaluation, clinical examination and anthropometric measurements, biochemical and serological tests. The cut-off values for central obesity were waist hip ratio (WHR) > 0.85 in women and > 0.9 in men.

**Results:**

The prevalence of NAFLD was highest in the age group of 31 to 60 years. It was more common in males than females. Twenty cases (11.7%) had discomfort at right upper abdomen. Hepatomegaly was found in 27 patients (13.2%), impaired glucose tolerance (IGT) in 29 (14.21%) and diabetes mellitus in 38 (18.63%) patients. Overweight or obesity was found in 110 (53.92%) cases and central obesity was seen in 129 (63.23%) patients. Hence, metabolic syndrome (according to International Diabetes Federation Criteria) was present in 62.25% cases of NAFLD. Alanine aminotransferase (ALT) more than upper limit of normal was found in 36.76% cases.

**Conclusion:**

Risk factors for NAFLD in Bangladesh are similar to reported from the rest of the world. Age more than 30 years, male sex, WHR > 0.9 in men and more than 0.85 in female, BMI more than 25, glucose intolerance are predictive factors for NAFLD.

**How to cite this article:**

Shil BC, Saha M, Ahmed F, Dhar SC. Nonalcoholic Fatty Liver Disease: Study of Demographic and Predictive Factors. Euroasian J Hepato-Gastroenterol 2015;5(1):4-6.

## INTRODUCTION

Nonalcoholic fatty liver disease (NAFLD) represents a spectrum of liver disease characterized by excess of fat in liver ranges from simple steatosis to nonalcoholic steatohepatitis (NASH), cirrhosis and hepatocellular carcinoma (HCC) in the absence of secondary causes of hepatic fat accumulation.^1,2^ Nonalcoholic fatty liver disease is the most common liver disorder in western industrialized countries, affecting 20 to 30% of the general population and recent studies indicate that fatty liver is an emerging problem in the Asia-pacific region affecting.^3-5^ The predisposing factors for NAFLD are metabolic syndrome, diabetes mellitus, obesity, insulin resistance and dyslipidemia.^6,7^ It is becoming a major public health problem due to advent of increasingly sedentary life styles, changing dietary patterns, rising incidence of obesity and type 2 diabetes mellitus.^5^ Nonalcoholic fatty liver disease may contribute to cardiovascular disease through the release of proinflammatory mediators that damage the endothelium.^8^ Nonalcoholic fatty liver disease/ nonalcoholic steatohepatitis is now considered to be common cause of chronic liver disease with higher risk of processing to hepatocellular carcinoma (HCC) and an increasing indication for liver transplantation in western countries.^9^

Most patients with NAFLD are asymptomatic and typically with moderate elevation in transaminases (ALT or AST); the ALT/AST ratio is usually less than 1.010. Ultrasonography has 85 to 93% sensitivity and specificity to diagnose fatty liver. The only definite diagnostic investigation for NAFLD/NASH is liver biopsy but disadvantages to biopsy include observer variability, sampling variation and morbidity and mortality.^9,10^ Fibroscan is a new noninvasive imaging modality to detect the liver fibrosis.^9^

Published data on NAFLD from Bangladesh are very few. This study was carried out to find out the demographic and predictive factors associated with NAFLD.

## MATERIALS AND METHODS

The study was carried out in three different districts and at three centers of Dhaka city. It includes both urban and rural population from January 2011 to December 2012. Number of study population was 216. All the subjects with sonologically defined fatty liver and underwent detailed history evaluation, clinical examination and anthropometric measurements, and biochemical and serological tests. Fatty liver was defined by the presence of at least two of three abnormal findings on abdominal ultrasound: (1) diffusely increased echogenicity (bright) liver with liver echogenicity greater than kidney with (2) vascular blurring and (3) deep attenuation of ultrasound signal. Persons with alcohol intake, seropositivity for HBV or HCV, other known liver disease and taking medication causing liver disease were excluded. The cut-off values for body mass index (BMI) was based on accepted criteria. The cut-off values for central obesity were waist hip ratio > 0.85 in women and > 0.9 in men.

## RESULTS

Total 216 individuals with sonologic fatty liver disease were initially enrolled for the study. Five patients consumed alcohol, four patients were positive for HBsAg, two patients were expressing anti-HCV and one patient was taking steroids; all of them were excluded from final analysis; thus, the study analyzed 204 patients. The numbers of males were 118 and females were 86. The demographic data as well as clinical features and biochemical data have been shown in Table 1.

**Graph 1:**
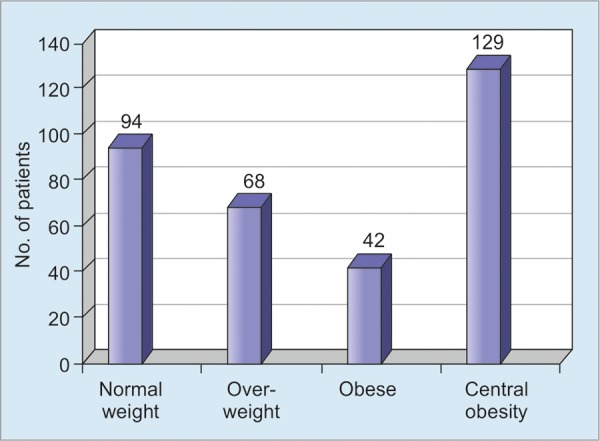
Body mass index and central obesity in NAFLD patients

The status of obesity in the study population is shown in Graph 1.

## DISCUSSION

In our study, we have examined sonologic fatty liver cases attending out patient department (OPD) of six centers to find out the demographic and predictive factors of NAFLD. It is reasonable to believe that at least a small proportion of patients with sonologically normal liver may still be having NAFLD as it is known that specificity and sensitivity of USG in identifying fatty liver is 85 to 93% only.^9,11^

Nonalcoholic fatty liver disease was found commonly in 31 to 60 years of age group with a male predominance. Studies in India suggested similar results.^7,9^ In this study, we found that NAFLD presented with mild right upper abdominal pain, dyspepsia and mild hepatomegaly which also coincided with findings of Duseja A.^9^

Obesity and in particular central obesity have been described as one of the strongest risk factors for NAFLD.^1,2^ In our study, prevalence of overweight (33.3%), obesity (20.6%) and central obesity (63.2%) was also seen in high percentage. Study from Delhi has found similar obser-vation.^12^

Nonalcoholic fatty liver disease has been associated very closely with the presence of type 2 diabetes mellitus. Diabetes mellitus is an important determinant of both presence and severity of NAFLD.^1,2,8^ In this study, we found impaired glucose tolerance and diabetes mellitus in 14.21 and 18.63% patients respectively.

**Table 1:** **Table 1:** Demographic data, clinical features and biochemical parameters of study population

*Parameters*		*Value*		*Percentage*	
Age in years		36.2 ± 10.3		—	
Gender (male:female)		1.37:1		—	
Right upper abdominal pain		20		11.76	
Dyspepsia		45		22.05	
Hepatomegaly		27		13.23	
Diabetes mellitus		38		18.63	
Hypertension		32		15.69	
IHD		28		13.73	
Raised ALT		75		36.76	
Raised AST		28		13.72	
Raised total cholesterol		125		61.27	
Raised LDL		105		51.47	
Low HDL		110		53.92	
Raised triglycerides		121		59.31	
Metabolic syndrome		127		62.25	
Gallstone disease		12		5.88	

Hypertension has also been reported frequently in patients with NAFLD^11^ but it was observed in 15.69% of patients in our study and that is comparable with that from India.^9^ Dyslipidemia has been reported in 20 to 92% of patients of NAFLD;^1,2,11^ in our study, it was present in more than 50% of patients which is similar with the finding reported by of Duseza A.^9^

Nonalcoholic fatty liver disease appears to be the hepatic manifestation of metabolic syndrome.^6^ It was observed in 62.25% patients of our study which also well coincided with findings of other studies.^9,11^ Prevalence of gallstones was 5.88% which is almost similar with previous study of our country (6%) and study at USA (7.4%).^13^
